# Pregnancy outcomes in women with repeated implantation failures after intracytoplasmic morphologically selected sperm injection (IMSI)

**DOI:** 10.1186/1477-7827-9-99

**Published:** 2011-07-22

**Authors:** João Batista A Oliveira, Mario Cavagna, Claudia G Petersen, Ana L Mauri, Fabiana C Massaro, Liliane FI Silva, Ricardo LR Baruffi, Jose G Franco

**Affiliations:** 1 Department of Gynecology and Obstetrics, Botucatu Medical School Sao Paulo State University - UNESP, Botucatu, Brazil; 2 Paulista Centre for Diagnosis, Research and Training, Ribeirao Preto - SP, Brazil; 3 Centre for Human Reproduction, Prof. Franco Jr., Ribeirao Preto, Brazil; 4 Postgraduate Fellow Department of Gynecology and Obstetrics, Botucatu Medical School Sao Paulo State University - UNESP, Brazil

## Abstract

**Background:**

The purpose of this study was to compare laboratory and clinical outcomes of intracytoplasmic morphologically selected sperm injection (IMSI) and conventional intracytoplasmic sperm injection (ICSI) in couples with repeated implantation failures.

**Methods:**

A total of 200 couples with at least two prior unsuccessful ICSI cycles were enrolled: 100 couples were submitted to IMSI and 100 were submitted to routine ICSI. For IMSI, spermatozoa were selected at 8400× magnification using an inverted microscope equipped with Nomarski (differential interference contrast) optics. For conventional ICSI, spermatozoa were selected at 400× magnification. Clinical outcomes were evaluated between the two groups.

**Results:**

Study patients were comparable in age, number of treatment failures, aetiology of infertility, percentage of normal form assessed by MSOME (motile sperm organelle morphology examination), semen parameters, total number of oocytes collected, number of mature oocytes collected, total number of embryos transferred and number of high-quality embryos transferred. No statistically significant differences between the two groups were observed with regard to rates of fertilisation, implantation and pregnancy/cycle. Although not statistically significant, rates of miscarriage (IMSI:15.3% vs ICSI:31.7%), ongoing pregnancy (IMSI:22% vs ICSI:13%) and live births (IMSI:21% vs ICSI:12%) showed a trend towards better outcomes in the IMSI group. In addition, analysis of subpopulations with or without male factor showed similar results.

**Conclusions:**

Our results suggest that IMSI does not provide a significant improvement in clinical outcome compared to ICSI, at least in couples with repeated implantation failures after conventional ICSI. However, it should be noted that there were clear trends for lower miscarriage rates (≈50% reduced) and higher rates of ongoing pregnancy and live births (both nearly doubled) within the IMSI group. Further confirmation as well as randomized large-scale trials are needed to confirm the beneficial effects of IMSI in couples with poor reproductive prognoses.

## Background

Implantation failure is the major cause negatively influencing the outcome of assisted reproductive technologies (ART), as only two out of every ten embryos successfully implant [1]. The outcome of intracytoplasmic sperm injection (ICSI) has been shown to be positively associated with the morphological state of the sperm [2-7], while early miscarriage rates were negatively associated with nuclear morphology [5-7]. However, repeated failure of conventional IVF has been suggested to be caused by a paternal effect on early embryo development, a hypothesis confirmed using a shared oocyte donation model [8-11].

Bartoov et al. [5,12] developed a method of human spermatozoa evaluation performed in real-time at high magnification called "motile sperm organelle morphology examination" (MSOME). MSOME is performed using an inverted microscope equipped with Normarski interference contrast optics, which enables observation at high magnification (> 6000×) compared to the 200-400× observed by conventional ICSI [12]. This method led to the development of the intracytoplasmic morphologically selected sperm injection (IMSI) procedure, which is based on sperm normality as defined by MSOME classification and aims to improve conventional ICSI outcomes by focusing on the correlation between abnormalities in sperm morphology observed at high magnification and DNA damage [5,13-20]. Various studies have demonstrated that IMSI significantly improves fertilisation rates [21,22], embryo quality [5,11,20,21,23], the rate of development up to the blastocyst stage [21,24], the rates of implantation and pregnancy after embryo transfer on day 2 or 3 [5-7,11,13,20,22,23,25,26] or in the blastocyst stage [24,27] and the likelihood of having a normal healthy child [28]; IMSI also appears to significantly decrease miscarriage rates [5-7,13,23,26,29]. In fact, prior failures in ICSI cycles constituted an inclusion criterion in several studies employing IMSI [6,13,24,28,30].

To better comprehend the value of IMSI/MSOME, the purpose of this study was to compare laboratory and clinical outcomes of IMSI versus conventional ICSI in couples with repeated ICSI failures.

## Materials and methods

### Study participants

A total of 200 couples with at least two prior implantation failures were prospectively evaluated. Patients were divided into two groups matched by (female) age:

Group ICSI: 100 patients submitted to conventional ICSI.

Group IMSI: 100 patients submitted to IMSI.

All couples enrolled in the study met the following inclusion criteria: subfertility with a need for assisted reproduction with ejaculated spermatozoa, two or more failed ICSI attempts with embryos of good morphological quality (four identical blastomeres isolated 44 h after sperm injection with no fragments or multinucleation present [31-33]), a maternal age ≤39 years, a normal karyotype in both partners and negative for uterine defects, ultrasonographic evidence of hydrosalpinx, evidence of low ovarian response in previous treatment cycles (less than four oocytes retrieved), infections, endocrine problems, coagulation defects or thrombophilia and autoimmune defects (including antiphospholipid antibodies). The division between groups was performed as follows: after referral of the patient for IMSI, the subsequent patient with the same characteristics (i.e., least two implantation failures, same age (± 1 year) and fulfilled the inclusion criteria) was referred for ICSI. Written informed consents were obtained from all couples, and this study was approved by an internal institutional review board.

### Ovarian stimulation and oocyte recovery culture protocol

All patients were submitted to the same scheme of controlled ovarian stimulation. First, pituitary downregulation was established with nafarelin acetate at a dose of 400 mg/day (Synarel^®^; Pfizer, Brazil) started during the luteal phase of the previous cycle. After 14 days of treatment with the GnRH analogue and after a confirmation of downregulation, the administration of recombinant FSH (Gonal F^®^; Serono Barueri, SP, Brazil) was initiated at a dose of 150-250 IU, depending on patient age, and 75 IU/day of recombinant LH (Luveris^®^, Serono, SP, Brazil) for a period of 7 days. When one or more follicles measuring ≥17 mm were observed, recombinant HCG (Ovidrel^®^; Serono, Brazil) was administered at a dose of 250 μg, and oocyte retrieval was scheduled 35-36 hours later.

### Semen preparation

Semen samples were collected in sterile containers by masturbation after a sexual abstinence period of 2 to 5 days. The liquefied, fresh semen samples were prepared using ISolate (Irvine Scientific, Santa Ana, CA, USA) discontinuous concentration gradient. The final pellet was resuspended in 0.2 mL of modified HTF medium (Irvine Scientific, Santa Ana, CA, USA) supplemented with 10% human serum albumin (Irvine Scientific, Santa Ana, CA, USA).

Each semen sample was analysed for morphology according to the MSOME criteria [12] and for standard semen quality parameters according to the World Health Organisation[34].

### ICSI procedure

Conventional ICSI was performed using a Nikon Eclipse TE 300 inverted microscope equipped with Narishige 231 D-2 (Narishige, Tokyo, Japan) remote control hydraulic micromanipulators and Narishige IM-9B injectors. Spermatozoa were selected at 400× magnification using Hoffman modulation contrast following a set of published guidelines.

### IMSI procedure

A 1-μL aliquot of sperm cell suspension was transferred to a 5-μL microdroplet of modified HTF medium containing 7% polyvinylpyrrolidone solution (PVP medium Irvine Scientific, USA). The microdroplet was placed into a sterile glass dish (FluoroDish™ Word Precision Instrument, USA) under sterile paraffin oil (Ovoil-100, Vitrolife, Goteborg, Sweden). Sperm cells suspended within the microdroplet were placed on a microscope stage above an Uplan Apo 100 oil/1.35 objective lens previously covered by a droplet of immersion oil. In this manner, suspended motile sperm cells in the observation droplet could be examined at high magnification through an inverted microscope (Eclipse TE 2000 U Nikon, Japan) equipped with high-powered differential interference contrast optics (DIC/Nomarski). Images were captured using a colour video camera containing effective picture elements (pixels) for high-quality image production and projected onto a colour video monitor. Morphological evaluation was accomplished using a monitor screen, and the total calculated magnification was 8400× (total magnification: objective magnification = 100×, magnification selector = 1.0×, video coupler magnification = 1.0×, calculated video magnification = 84.50×).

A spermatozoon used for IMSI was classified as morphologically normal if it exhibited a normal nucleus as well as an acrosome, a post-acrosomal lamina, a neck and a tail, and did not present a cytoplasmic droplet or cytoplasm around the head [12]. The subcellular organelles were morphologically classified based on the presence of specific malformations, which were defined according to the arbitrary descriptive approach reported by Bartoov et al. [12] after studies utilising transmission and scanning electron microscopy. According to transmission electron microscopy estimations [6,12], a morphological normal state for the nucleus was defined by the shape and content of the chromatin. The criterion for normality of nuclear form was a smooth, symmetric and oval configuration. Normal means for length and width were estimated as 4.75 ± 2.8 and 3.28 ± 0.20 μm [12], respectively, and the form was classified as abnormal when it presented a variation of 2 standard deviations in one of the axes (length: ≥5.31 or ≤4.19 μm; width: > 3.7 or < 2.9 μm). For rapid evaluation of nuclear form, a fixed, transparent, celluloid form of a sperm nucleus fitting the criteria was superimposed on the examined cell (chablon construction based on ASTM E 1951-2 [35]). In the same manner, the nuclear form was considered abnormal if extrusion or invagination of the nuclear chromatin mass was detected (regional malformation of nuclear form). Chromatin content was considered abnormal if one or more vacuoles were observed to occupy more than 4% of the nuclear area (visual evaluation was aided by a celluloid form of a large vacuole superimposed on the examined cell, if necessary). A nucleus was considered normal if both nuclear form and chromatin content were normal.

The same technician performed all sperm selection, and only normal spermatozoa were injected in this study. The time involved in the selection step was 30-120 min/sample, and at least three spermatozoids were selected for each MII oocyte. After sperm selection, microinjections were carried out in the same manner as in ICSI. Spermatozoids were still motile when captured for final selection.

### Oocyte and embryo culture and transfer

Sperm-injected oocytes, zygotes and embryos from both IMSI and ICSI groups were submitted to the same culture conditions. On day 2, the two/three best-scoring embryos from both ICSI and IMSI groups were transferred. The quality of transferred embryos was similar between the groups.

### Statistical analysis

Data were analysed using InStat version 3.0 (GraphPad Software, San Diego California, USA) on a Macintosh computer (Apple Computer Inc., Cupertino, California, USA). The primary endpoint of the investigation was the ongoing pregnancy rate and secondary endpoints were the rates of fertilisation, clinical pregnancy per cycle, miscarriage and live births. Fertilisation was defined as zygotes containing two pronuclei after ICSI or IMSI. Pregnancy was defined as the presence of a gestational sac with heartbeat visualized by ultrasound 4-6 weeks after embryo transfer. Miscarriage was defined as the termination of the pregnancy up to 20 weeks of gestation. The variables were analysed in relation to the general population as well as the subpopulations with or without male factor. The sample size for the general study was calculated by comparing between two proportions: control (ICSI cycles) and experimental (IMSI cycles). In general, the mean ongoing pregnancy rate was 25%. Thus, a sample size of 100 patients in each group had 80% power to detect an increase of 20% with an alpha significance level of 0.05 (two-tailed). When appropriate, Student's *t*-tests, Mann-Whitney tests and chi-square tests were utilised with a significance level of *P *< 0.05.

## Results

The characteristics of the study patients (general population) in both the IMSI and ICSI groups were comparable with regard to age, the number of treatment failures, aetiology of infertility, the percentage of normal form assessed by MSOME, semen parameters, the total number of oocytes collected, the number of mature oocytes collected, the total number of embryos transferred and the number of high-quality embryos transferred. No statistically significant differences (*P *> 0.05) were observed between the two groups with regard to rates of fertilisation (IMSI: 65.4 ± 23.5%; ICSI: 62 ± 26.5%), implantation (IMSI:13.6%; ICSI:9.8%) and pregnancy/cycle (IMSI:26%; ICSI:19%). Although not statistically significant, rates of miscarriage (IMSI:15.4%; ICSI:31.6%), ongoing pregnancy (IMSI:22%; ICSI:13%) and live births (IMSI:21%; ICSI:12%) displayed a clear trend towards better outcomes in the IMSI group. Table 1 summarises the characteristics and outcomes of the general study population.

**Table 1:** General study population; comparison between morphologically selected sperm injection (IMSI) and conventional intracytoplasmic sperm injection (ICSI) groups.

	Total		
	IMSI	ICSI	*P*
Cycles (*n*)	100	100	
Female age (years)	36.8 ± 3.9	36.7 ± 4.0	0.80
Male age (years)	39.8 ± 6.2	40.3 ± 6.0	0.50
Number of failures	3.3 ± 1.7	3.2 ± 1.6	0.49
Aetiology (%)			
Male	29	35	0.12
Idiopathic	24	17	
Tuboperitoneal	19	14	
Endometriosis	18	18	
Male + endometriosis	6	2	
Tuboperitoenal+ endometriosis	2	6	
Male+tuboperitoneal	2	8	
MSOME ^a^			
Normal spermatozoa (%)	1.5 ± 1.8	1.7 ± 1.8	0.32
Semen Parameters ^b^			
Total sperm count (x10^6^/mL)	70.4 ± 83.4	70.7 ± 60.4	0.97
Motility (% spermatozoa) (rapid + slow progression)	51.8 ± 19.7	49.6 ± 23.7	0.55
Vitality (%) (mean ± SD)	60.5 ± 17.4	56.0 ± 16.9	0.20
Leukocytes in semen (x10^6^) (mean ± SD)	0.4 ± 0.6	0.5 ± 0.4	0.22
Number of oocytes			
Mature (MII)	7.1 ± 4.8	6.7 ± 3.5	0.94
Total	9.5 ± 6.2	8.4 ± 4.6	0.45
Fertilisation rate (%)	65.4 ± 23.5	62 ± 26.5	0.34
Embryos transfer (*n*)	2.7 ± 1.0	2.7 ± 1.0	0.44
High-quality embryo transfer (*n*)	1.4 ± 0.5	1.5 ± 0.5	0.53
Implantation rate (%)	13.6	9.8	0.21
Pregnancy/cycle (%)	26	19	0.73
Miscarriage (%)	15.4	31.6	0.28
Ongoing pregnancy/cycle (%)	22	13	0.13
Live birth/cycle (%)	21	12	0.12

^a ^motile sperm organelle morphology examination

^b ^semen quality parameters according to the World Health Organisation[34]

In the subpopulation of cases with male factor (IMSI: 37 couples; ICSI: 45 couples) characteristics of study patients were comparable with regard to age, the number of treatment failures, the percentage of normal form assessed by MSOME, semen parameters, the total number of oocytes collected, the number of mature oocytes collected, the total number of embryos transferred and the number of high-quality embryos transferred. Similar to the general population, no statistically significant differences (*P *> 0.05) were observed between the two groups with regard to rates of fertilisation (IMSI: 59.1 ± 23.1%; ICSI: 57.8 ± 25.2%), implantation (IMSI: 11.8%; ICSI: 11.2%) and pregnancy/cycle (IMSI: 32.4%; ICSI: 24.4%). Rates of miscarriage (IMSI: 16.7%; ICSI: 36.4%), ongoing pregnancy (IMSI:27%; ICSI: 15.5%) and live births (IMSI:27%; ICSI: 15.5%) again showed a clear trend towards better outcomes in the IMSI group, although they were not considered statistically significant. These results are summarised in Table 2.

**Table 2:** Subpopulations with or without male factor; comparison between morphologically selected sperm injection (IMSI) and conventional intracytoplasmic sperm injection (ICSI) study groups.

	With male factor			Without male factor		
	IMSI	ICSI	*P*	IMSI	ICSI	*P*
Cycles (*n*)	37	45		63	55	
Female age (years)	36.9 ± 4.0	35.6 ± 3.9	0.11	36.6 ± 4.0	37.7 ± 3.9	0.13
Male age (years)	40.6 ± 6.4	41.9 ± 6.5	0.34	39.3 ± 6.0	39.0 ± 5.3	0.79
Number of failures	3.1 ± 1.6	3.4 ± 1.9	0.64	3.4 ± 1.7	3.0 ± 1.3	0.20
Aetiology (%)						
Male	78.4	77.8	0.08			0.33
Idiopathic	0	0		38	30.9	
Tuboperitoneal	0	0		30.2	25.4	
Endometriosis	0	0		28.6	32.7	
Male + endometriosis	16.2	4.4		0	0	
Tuboperitoenal+ endometriosis	0	0		3.2	11	
Male+tuboperitoneal	5.4	17.8		0	0	
MSOME ^a^						
Normal spermatozoa (%)	0.3 ± 0.3	0.3 ± 0.3	0.79	2.4 ± 1.9	2.5 ± 2.1	> 0.99
Semen Parameters						
Total sperm count (x10^6^/mL)	14.2 ± 18.4	11.8 ± 14.9	0.86	95.8 ± 88.9	98.9 ± 52.5	0.82
Motility (% spermatozoa)	35.5 ± 14.1	29.6 ± 15.6	0.12	60.9 ± 16.2	61.1 ± 10.8	0.94
(rapid+slowprogression)	49.6 ± 15.8	47.2 ± 15.2	0.54	65.5 ± 15.9	70 ± 6.3	0.56
Vitality (%) (mean ± SD)	0.3 ± 0.3	0.4 ± 0.5	0.21	0.4 ± 0.8	0.5 ± 0.3	0.16
Leukocytes in semen (x10^6^) (mean ± SD)						
Number of oocytes						
Mature (MII)	8.0 ± 5.1	7.8 ± 3.7	0.82	6.6 ± 4.4	5.9 ± 3.1	0.56
Total	10.7 ± 6.8	9.4 ± 4.6	0.61	8.7 ± 5.7	7.6 ± 4.4	0.44
Fertilisation rate (%)	59.1 ± 23.1	57.8 ± 25.2	0.61	68.1 ± 24.4	65.4 ± 27.4	0.72
Embryos transfer (*n*)	2.6 ± 1.0	2.8 ± 1.1	0.69	2.7 ± 1.1	2.6 ± 1.0	0.63
High-quality embryo transfer (*n*)	1.5 ± 0.5	1.5 ± 0.5	0.89	1.4 ± 0.5	1.5 ± 0.5	0.55
Implantation rate (%)	11.8	11.2	0.88	14.6	8.6	0.16
Pregnancy/cycle (%)	32.4	24.4	0.57	22.2	14.5	0.40
Miscarriage (%)	16.7	36.4	0.54	14.3	25	0.95
Ongoing pregnancy/cycle (%)	27	15.5	0.31	19	10.9	0.33
Live birth/cycle (%)	27	15.5	0.31	17.5	9.1	0.29

^a ^motile sperm organelle morphology examination

^b ^semen quality parameters according to the World Health Organisation[34]

In relation to the subpopulation without male factor (IMSI: 63 couples; ICSI: 55 couples), there were no differences in patient age, the number of treatment failures, the percentage of normal form assessed by MSOME, semen parameters, the total number of oocytes collected, the number of mature oocytes collected, the total number of embryos transferred and the number of high-quality embryos transferred between the two groups. Once again, no differences (*P *> 0.05) were observed in rates of fertilisation (IMSI: 68.1 ± 24.4%; ICSI: 65.4 ± 27.4%), implantation (IMSI: 14.6%; ICSI: 8.6%) or pregnancy/cycle (IMSI: 22.2%; ICSI: 14.5%). As in previous analyses, rates of miscarriage (IMSI: 14.3%; ICSI: 25%), ongoing pregnancy (IMSI: 19%; ICSI: 10.9%) and live births (IMSI: 17.5%; ICSI: 9.1%) displayed a clear trend towards better outcomes in the IMSI group, although they were not statistically significant. These results are summarised in Table 2.

## Discussion

The aim of ART is to birth a single, healthy baby, and the success of this event depends on many variables. Despite advances in the field of assisted reproduction, implantation rates have not improved significantly since the ICSI technique was established in 1992 [36]. It is well known that implantation rates are related to endometrial receptivity, cytokines, and growth factors secreted by ovarian stimulation [37-39]. Poor embryo quality is another important factor that negatively affects implantation rates [40]. Choosing sperm using MSOME parameters appears to provide better rates of fertilisation and embryo quality [11,21], which also translates to better conditions during the implantation process. It is well established that the capacity of human sperm to fertilise an oocyte and produce an embryo with high potential of implantation and development depends, among other factors, on DNA integrity [15,16]. Observations from our group have shown that the presence of large nuclear vacuoles (LNV), which are sperm-specific alterations observed at high magnification, may be related to DNA fragmentation and denaturation [15]. Vanderzwalmen et al. [24] also reported a negative impact of the presence of one large nuclear vacuole or several small vacuoles in the head of spermatozoa on the competence of embryos to develop to the blastocyst stage. Moreover, Wilding et al. [20] had suggested that a correlation exists between LNV and DNA fragmentation. Even if percentages of fertilisation and embryo development are comparable to those of conventional ICSI, embryos obtained from spermatozoa analysed by MSOME showed higher quality, and more overall pregnancies were achieved. On the other hand, recent investigations have related LNV to the absence of an acrosome reaction, which could explain the hampering of embryo development when a spermatozoon with this characteristic is chosen for microinjection [41]. Gopalkrishnan *et al*. [42] found that the chromatin of spermatozoa from men whose partners presented with early pregnancy loss was often compact or partially compact with irregular nuclear borders and large vacuoles.

Currently, ICSI is performed after morphological selection of spermatozoa at 200× to 400× magnification. In this magnification range, spermatozoa carrying defects of the head, neck or tail can be detected, but nuclear vacuoles cannot. Therefore, observation at a magnification of 6000× to 12,500× when selecting spermatozoa appears to be better by simply allowing precise identification of vacuoles [6,7,15,16,20,23,24]. Nevertheless, in couples with repeated implantation failures, sperm selection at high magnification does not appear to exert much significant influence. Fertilisation rates were similar between the IMSI and ICSI groups, a result not influenced by the presence or absence of male factor, and confirms other prior findings that also did not detect any significant differences in fertilisation rates [5,11,12,23]. Regarding clinical results, there was again no significant influence of sperm selection at high magnification both in the general population and in subgroups with or without male factor. However, there was a significant and clear trend for better results in the IMSI group, particularly for rates of ongoing pregnancy, live births (both nearly doubled) and miscarriage (≈50% reduced). Although not statistically significant (likely due to sample size), these results can be considered clinically relevant. Given that apparently normal spermatozoa visualized in the low magnification range in conventional ICSI may possess hidden anomalies that could be detected at higher magnification with MSOME, the use of sperm selected by IMSI can produce embryos with higher developmental capacity. This would exclude using sperm with higher probabilities of DNA fragmentation and denaturation.

This trend is in agreement with several published studies. In addition, other investigations reported that IMSI is able to increase pregnancy rates and reduce miscarriage rates in assisted reproduction cycles [5,11,14,20,23,26]. In a prospective randomized study comparing the clinical outcome of 87 IMSI cycles with 81 conventional ICSI cycles, Balaban et al. [25] reported that IMSI did not provide any significant improvement in the clinical outcome compared to ICSI. However, there were trends for higher rates of implantation (28.9% versus 19.5%), clinical pregnancy (54.0% versus 44.4%) and live births (43.7% versus 38.3%) in the IMSI group. On the other hand, in this same study [25], severe male factor patients were found to benefit from the IMSI procedure, as significantly higher implantation rates were observed compared to their counterparts in the ICSI group.

In conclusion, the results obtained in this investigation suggest that IMSI does not appear to provide a significant improvement in clinical outcome compared to ICSI, at least in couples with repeated implantation failures after conventional ICSI. However, it should be noted that there were clear trends for lower rates of miscarriage (≈50% reduced) and higher ongoing pregnancy and live births (nearly doubled) in the IMSI group. Thus, using morphologically-selected spermatozoa with IMSI would benefit those with poor reproductive prognosis; however, further confirmation in addition to randomized large-scale trials are still needed.

## Conflicts of Interest

The authors declare that they have no competing interests.

## Authors' contributions

JBAO designed and coordinated the study. All authors were responsible for data collection, analysis, and interpretation presented in the manuscript. JBAO, MC, RLRB and JGF performed the statistical analyses and wrote the manuscript; JGF reviewed the manuscript. All authors read and approved the final manuscript.
